# Systems thinking with active implementation research (STAIR): Protocol for a school-based randomised control trial for childhood obesity prevention

**DOI:** 10.1371/journal.pone.0325853

**Published:** 2025-06-24

**Authors:** Michelle Jackson, Jillian Whelan, Steven Allender, Luke Wolfenden, Melanie Nichols, Vicki Brown, Sze Lin Yoong, Liliana Orellana, Kristy A. Bolton, Penelope Love, Prabhat Lamichhane, Nicole Nathan, Colin Bell

**Affiliations:** 1 Deakin University, School of Medicine, Global Centre for Preventive Health and Nutrition, Institute for Health Transformation, Geelong, Australia; 2 Deakin University, School of Health and Social Development, Global Centre for Preventive Health and Nutrition, Institute for Health Transformation, Geelong, Australia; 3 Deakin University, School of Medicine, Geelong, Australia; 4 School of Medicine and Public Health, The University of Newcastle, Newcastle, Australia; 5 Deakin University, Deakin Health Economics, Global Centre for Preventive Health and Nutrition, Institute for Health Transformation, Geelong, Australia; 6 Deakin University, Biostatistics Unit, Faculty of Health, Geelong, Australia; 7 Deakin University, Institute for Physical Activity and Nutrition, Geelong, Australia; 8 Department of Public Health, La Trobe University, Melbourne, Australia; 9 National Centre of Implementation Science, The University of Newcastle, Newcastle, Australia; PLOS: Public Library of Science, UNITED KINGDOM OF GREAT BRITAIN AND NORTHERN IRELAND

## Abstract

**Introduction:**

This paper presents the protocol for the effectiveness-implementation trial: Systems Thinking with Active Implementation Research (STAIR) for childhood obesity prevention. STAIR’s objective is to protect primary school children in Victoria, Australia from unhealthy weight and test implementation strategies for sustaining healthy school environments. STAIR is a 3-year cluster-randomised controlled trial with repeat-cross-sectional child-level data collection utilising an effectiveness-implementation type-1 hybrid design. Schools will be recruited from non-metropolitan government primary schools in Southwest Victoria, Australia, with consenting schools (target = 28) randomly assigned (1:1) to intervention or control, and students in grades 1–6 (n = 3000–6000) recruited using an opt-out approach.

**Intervention:**

The intervention, co-designed by each school community, will involve multi-step group model building and action formulation process using community-based systems dynamics and baseline data (i.e., child weight status, HRQoL, health behaviours, and school environment characteristics). Implementation strategies will be guided by an active implementation framework, and coordinated by a leadership team of school representatives, community partners, and researchers.

**Primary and secondary outcome measures:**

Primary outcomes are to measure (1) change in standardised body mass index (z-BMI) between intervention and control groups after three year and (2) change in acceptance and adoption of STAIR interventions and support. Secondary outcomes include differences in obesity prevalence, health behaviour measures and HRQoL, between intervention and control groups after three years. Implementation outcomes will focus on strategy effectiveness, acceptability, and utilisation. Secondary data will be collected from schools and partner organisations. An economic evaluation will assess the cost-effectiveness of intervention and implementation support.

**Conclusion:**

STAIR aims to create lasting changes in schools to prevent childhood obesity by supporting healthy environments and providing insights into interventions’ cost-effectiveness and feasibility. The findings will guide policymakers and educational institutions in adopting sustainable health promotion strategies.

**Trial registration:**

ANZCTR-ACTRN12624000461594p

## Introduction

The case for action to prevent childhood obesity is compelling [1]. One in 4 children in Australia experience overweight or obesity. Prevalence has steadily increased from the 1980s to the 2000s and is unequally distributed socio-economically and geographically [2]. Children with obesity are stigmatised because of their weight, have a lower quality of life [1,3], have lower mastery of movement skills, and have more physical and psychosocial disability than their healthy-weight peers [4–6]. Unhealthy diets and insufficient physical activity are risk factors for child obesity, and obesity itself, endures into adulthood, putting children with obesity at risk of chronic diseases, such as type 2 diabetes, later in life [7,8]. Furthermore, adults living with obesity experience higher medical expenses, discrimination, and worse jobs [9]. Living with obesity is the leading contributor to the burden of disease in Australia, and the total cost was estimated at $1280 (US$940) per capita in 2019 [10]. Reviews of strategies for managing and treating obesity in childhood indicate they have limited effectiveness and can be costly and invasive [11–13].

The UN Convention on the Rights of the Child indicates that ‘children have the right to the best health care possible, clean water to drink, healthy food and a clean and safe environment to live in’ [14]. Interventions have been implemented in different settings including schools to create a healthy and safe environment. The most recent Cochrane review on childhood obesity prevention found changing diet and activity levels, particularly in school settings, can be effective in reducing standardised BMI scores in children aged 6–12 years [15]. The review acknowledged that the complex and dynamic determinants of childhood obesity require a systems approach involving interventions targeted at different parts of the system, leading to coordinated policy initiatives across various government departments [16].

In Australia, the national obesity prevention strategy has adopted such an approach [17], and our group has contributed to evidence of effectiveness. The research team’s latest trials (WHO STOPS, 2015–2019) [18] and (RESPOND 2019–2024) [19], drew on community-based systems dynamics to co-develop intervention actions, in line with recommendations from the Lancet Commission on the Global Syndemic of Obesity, Undernutrition, and Climate Change [20]. Community-based systems dynamics offers significant advantages over earlier approaches to intervention design by providing a communal and visualised understanding of local causes of childhood obesity and the complex interactions between them, and by achieving community proprietorship of and engagement in, prevention strategies [21]. It enhances rather than replaces existing activities and communities can identify and intervene in elements of the system where they can have the most impact. The WHO STOPS study achieved a 4% reduction in overweight and obesity prevalence and improved health-related quality of life (HRQoL) in intervention communities compared to no change in controls in the first two years of the trial [22]. However, while HRQoL and dietary changes were sustained after 4-years, weight status changes, which were similar in magnitude to other studies [15] were not. Other community-based interventions have also seen intervention effects on weight dissipate over time [23], and we think the reason is waning implementation support.

A recent systematic review identified ten characteristics for sustained implementation of community-based interventions, including leadership, workforce development, community engagement, partnerships, communication, policy, adaption, evaluation, resourcing, and governance. In WHO STOPS, the two-year time point coincided with a reduction in support from the research team (removal of trained staff available to support implementation) [18]. We hypothesised that this reduction in implementation support was the primary reason the intervention effect was not sustained as community capacity was low (e.g., too few staff, competing priorities). An unpublished audit conducted by the research team among rural-based communities, including WHO STOPS [24] and RESPOND [25] communities, revealed that rural prevention services were understaffed in Victoria, with only 1.4 full-time equivalents for every 10,000 people. For comparison, Australia has 37 doctors for every 10,000 people. Further, support available to schools in Victoria is largely online. Wolfenden *et al* identified that the complexity of the environment the community-based health service partners operate in is not well understood (size and diversity of the workforce, types and levels of training; mix of public and private organisations involved; mix of local, state and federal governance and funding structures) [26]. These implementation factors are also poorly measured, in part because researchers haven’t known what to measure. Collectively these observations reveal a tension between the expectations of researchers and policymakers on communities and their capacity to implement interventions, coupled with a scarcity of data on implementation [27].

In the face of low implementation capacity and competing priorities (such as responding to COVID-19) and increasingly obesogenic environments (such as advertising of unhealthy food) [28,29] we contend that communities, and particularly schools, need implementation support to sustain healthy environments. Working with school communities rather than whole communities may help focus implementation support and minimise competing priorities. Also, the Lancet Commission encourages greater application of expert-facilitated (not owned) implementation [20]. Our research has demonstrated it is possible to create environments that protect children from obesity and others have shown that healthy weight can persist after children have participated in school-based interventions [30,31]. However, there is a paucity of research exploring whether system-wide changes to school environments can be sustained so that successive cohorts of children are protected from unhealthy weight gain.

The primary objective of this study is to protect primary school children in rural Victoria, Australia, from unhealthy weight and test implementation strategies for sustaining school environments that promote health and wellbeing. The research questions being addressed in this study are: (1) can Victorian school communities be supported to prevent unhealthy weight gain for 3 years and; (2) what are the optimal implementation strategies to support delivery and maintenance of the intervention in schools? It is hypothesised: (1) a reduction of 0.13 units in z-BMI will be achieved in intervention schools relative to controls after 3 years of intervention and; (2) that implementation strategies will be cost-effective, acceptable, utilised, appropriate, feasible, delivered as intended and maintained in intervention schools.

## Methods and analysis

### Conceptual framework

**Fig 1:**
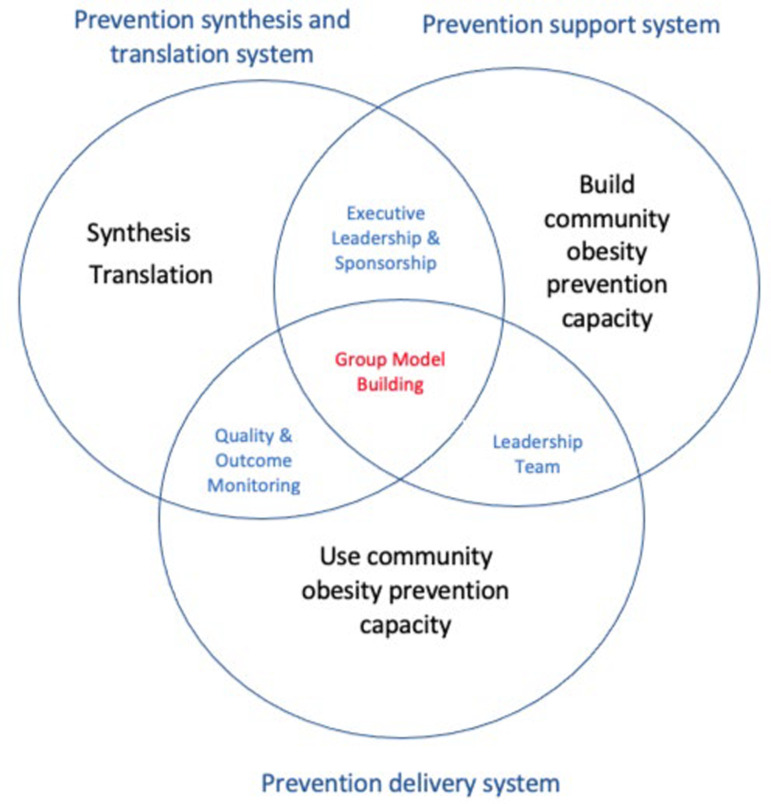
Conceptual framework.

Obesity prevention research is at the point where substantial knowledge about how to prevent childhood obesity is available but where that knowledge is not applied in practice [32]. The Interactive Systems Framework (ISF) was designed to facilitate the dissemination and implementation of such prevention knowledge. Given the gaps identified in implementation support for previous interventions, we used ISF to conceptualise a prevention support system (Fig 1). Obesity prevention will be the innovation we intend to disseminate and implement, school communities will be the prevention delivery system, and the research team will be the prevention synthesis and translation system (Fig 1). Trial partners, named below, are key stakeholders in this system. STAIR intends to strengthen prevention support and delivery systems (capacity in schools and among partners). Researchers in the field of applied implementation science have recognised the need to help communities translate prevention into practice and have developed frameworks and strategies to support this [33,34]. STAIR will use the Active Implementation Framework developed by Fixsen [35] to support schools so intended prevention outcomes (healthy weight, HRQoL) will be achieved and sustained. This framework was chosen as it emphasises system-wide change, active problem-solving, and on-going learning. It defines for the trial an executive and sponsorship function, a cross-system leadership team to coordinate and build overweight/obesity prevention capacity and quality, and outcome monitoring functions to ensure delivery of intended outcomes [36]. To complete the conceptual framework, we will place group model building (GMB), the co-design process from community-based systems dynamics [37], at the centre. Students, staff, and partners, will map the causes of childhood overweight/obesity and identify solutions and implementation strategies using GMB.

### Ethics and dissemination

Approval has been obtained from the Deakin University Human Research Ethics Committee (ID: 2024_051) and the Victorian Government Department of Education and Training (ID: 24-02-254). We had intended to recruit from Government and Catholic schools, but the Diocese of Ballarat Catholic Education Office did not approve the project, citing that the project did not align with the Diocese’s priorities, so we are continuing this study using Government schools.

### Partnerships and funding

STAIR is funded by the Australian National Health and Medical Research Council’s (NHMRC) Partnership Project Research Grant scheme (GNT2023737), supplemented by additional financial and in-kind contributions from four partner organisations who are signatories to the grant. Partners include Deakin University (sponsor), the Victorian Government Department of Education, the Royal Flying Doctor Service, VicHealth and the Victorian Government Department of Health.

### Trial design and status

**Fig 2:**
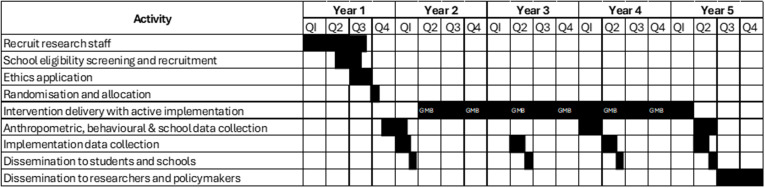
SPIRIT Schedule and timelines.

The STAIR project, conducted over five years with three intervention years, will comprise a cluster randomised controlled trial with repeat-cross sectional data collection, utilising an effectiveness-implementation type-1 hybrid design. Fig 2 shows the Standard Protocol Items: Recommendations for Intervention Trial (SPIRIT) schedule of enrolment, interventions, and assessments along with timelines (S1 Fig).

Participant recruitment and data collection have not begun. School recruitment for this study is planned to commence on 28/04/2025.

### Setting and recruitment

All government primary schools in southwest education region of Victoria excluding schools in the Brimbank, Melton and Western Melbourne education areas, schools participating in other overweight/obesity prevention interventions (as advised by Victorian Government Department of Education and Training), schools with fewer than 120 children and schools exclusively educating children with special needs will be eligible to participate in the trial. A randomly sorted list of all eligible Government schools (n = 97) will be generated using a random selection without replacement. Starting at the top of the list, school principals will be contacted by the research team, provided with information about the study and invited to provide written informed consent. Up to three follow-up phone calls and/or in-person visits will made to gain consent before moving on to the next school on the list. Recruitment will continue until 28 schools have consented (assuming a 33% recruitment rate). Previous studies in the region suggest school participation rates of 70% and student participation >80% [18].

### Randomisation and blinding procedure

After the full sample of schools is recruited schools will be randomly allocated (1:1), to intervention or control groups within strata defined by size (student enrolment ≥120). The study statistician will produce the random sequences and allocation will occur simultaneously for all schools. Given the nature of the intervention, it will be impossible to conceal the allocation from the school communities. However, data analysts will be blind to intervention allocation by removing school identification data.

### Children

The research team will visit all participating schools to give a brief presentation about the study to students in grades 1–6 (ages 6–12). They will distribute a plain language statement and an opt-out form for students to take home to their parents/guardians. Students will be included in data collection unless they or their parent/guardian actively opt-out. Surveys will be conducted at the start (baseline), and after the first, second, and third years of the intervention. All children present on the day of data collection who have not opted out will be eligible. Consequently, the composition of children included in each time point of data collection will vary, with some children being followed longitudinally, while others will not.

### Intervention

Before the commencement of the intervention, the research team will collect data on child weight status (using anthropometry), HRQoL, dietary behaviours, physical activity and sedentary behaviours, oral health (due to common risk factors with overweight/obesity [38]), sleep, and the school environment. The intervention process (Table 1) will comprise a series of facilitated GMB workshops (1–3 hours each) where this baseline data, along with evidence of best practice [33], will be provided to intervention school stakeholders (student leaders, teachers, parents, community leaders (including health promotion officers, general practitioners, journalists, food retailers) and partners). Student leaders will be children in grades 5 and 6 (aged approximately 10–12 years) as the Australian curriculum expects children by the end of grade 4 to use problem-solving skills to select and demonstrate strategies that help them stay safe, healthy, and active. Together, stakeholders will describe and visualise the causes of childhood overweight/obesity for the whole school community using causal loop diagrams employing Systems Thinking in Community Knowledge Exchange (STICKE) software [39]. Workshops will culminate in the formulation of specific actions the school community will take to create a healthier environment [40]. Communities find these methods engaging, effective and they translated to action faster than traditional approaches [18]. In previous trials, actions such as walking to school programs and improving the taste of the local water supply have been implemented to improve physical activity, healthy eating, children’s knowledge, and broader social determinants [41].

**Table 1:** Intervention process, purpose, actions, and frequency.

Intervention Activity	Purpose	Actions/Roles
**GMB workshop 1**	Problem mapping by student, school, and community leaders (n = 5–10)	Baseline data on BMI and wellbeing will be presented to school communities using an infographic and a facilitator (Prevention Support System Manager employed by the project) will use STICKE to help map the causes and consequences of overweight/obesity and student wellbeing in each school community.
**GMB workshop 2**	Problem development by student, school, and community leaders (n = 5–10)	The facilitator will use STICKE to help representatives further develop the map initiated in GMB1
**GMB workshop 3**	Action formulation, prioritisation and, leadership identification and readiness (n = 10–20)	Using the problem map, the facilitator will help participants identify existing actions, intervention points, new actions, school readiness for change and, leaders/champions for implementing the prioritised actions.

### Control

Control schools will receive the usual support available to Victorian schools including the Vic Kids Eat Well initiative introduced by the Victorian state health department [42] in 2022, aimed at establishing healthier food environments for children, along with the Victorian Healthy Schools Achievement Program [43]. This program is designed to aid schools in defining and attaining benchmarks related to healthy eating, physical activity, and mental well-being and may include health promotion support. Control schools will be provided with a written report summarising overweight/obesity prevalence, HRQoL, dietary behaviours, physical activity and sedentary behaviours, oral health, sleep, and the school environment at baseline, after each time-point of data collection, and at the end of the trial. At the end of the trial, they will also receive a report on the overall results of the STAIR trial and, should they wish to design their own intervention actions, access to the STICKE training modules and STICKE software.

### Implementation

After formulating intervention actions in the third GMB workshop, the STAIR implementation process comprises further GMB workshops at each school that will map intervention actions to existing, and in the pipeline, community infrastructure and resources and Active Implementation Framework functions (Table 2). These functions include a cross-system leadership team (self-nominated school representatives, researchers, and executive leaders/sponsors), a school implementation team and strengthening practitioner competence/confidence as well as quality and outcome monitoring. This implementation process is designed to overcome low implementation capacity and competing priorities for schools. It will identify how intervention actions will be implemented (implementation strategies), who will be involved in managing and completing actions and, where resourcing and accountability for various functions sit. Strategy definitions will be guided by the ERIC taxonomy (Expert Recommendations for Implementing Change) [33], MS teams will be used for communication and to foster collective learning within and between intervention schools. GMB workshops will be used to facilitate active problem-solving and adaptive intervention/implementation strategies [44].

**Table 2:** Active implementation functions, purpose, and frequency.

Implementation Functions	Purpose	Actions/Roles	Proposed Frequency
**Implementation strategy identification (GMB4)**	Mapping community infrastructure and integrating solutions	Leaders will identify implementation strategies that map the actions identified in GMB3 to existing community infrastructure and resourcing (current, in the pipeline) or to new resources.	1 (1 hr)
**Cross-system Leadership Team. Representatives from all schools, Research Team and partners**	Centralised leadership and coordination to build intervention school capacity and ensure alignment of activities and sharing of resources and infrastructure	Operationalise GMB4 map Create terms of reference and MOUs (resource allocation between schools and partners) Identify executive leaders/sponsors and guide executive (support system) actions Identify and provide leadership support for day-to-day leaders and student leaders Strategic communication and dissemination plans Responsible for intervention delivery support, risk management plan, community of practice	Monthly then Quarterly
**Executive leadership/ sponsorship (Partners)**	Create a supportive environment for intervention actions	Re-direct prevention system resources and identify opportunities for new resources targeted at schools identified needs Modify staff position descriptions Change the way community organisations work together Policy advice and feedback to the government	As above with additional meetings as required
**School Implementation Team** **Local, Day-to-Day leadership** **(School and student champions/ leaders)**	Implement intervention actions	Linked to LT Create and manage action plans Create readiness for new behaviours among stakeholders based on existing organisational readiness Develop trusted relationships with local school community stakeholders	Monthly meetings
**Practitioner competency and confidence (teachers and implementers)**	Ensure quality and delivery of intervention actions	LT develop duty statements and incentives for key roles that align with intervention actions LT to identify and provide training and continuing education opportunities for implementation teams	3-6 months
**Quality and outcome monitoring**	Ensure intended outcomes and maximise the benefits of the intervention actions	RT and LT to assess whether intervention actions are being delivered as intended RT to provide LT with collection systems (e.g., action register) and data (e.g., economic) on intervention actions to inform decision making	3-6 months
**Facilitation support (GMB5+)**	Ongoing learning Active problem-solving and adaptive solutions	Updates on outcomes and implementation will be presented using an infographic & a facilitator will use STICKE to help representatives modify map and update strategies	Every 6 months (2 hrs)

*RT=Research Team, LT=Leadership Team

### Outcomes

#### Primary intervention effectiveness outcomes.

The primary measure of intervention effectiveness will be the between-group difference in change over time from baseline to year 3 in childhood z-BMI score in repeat cross-sections of grade 1–6 children in intervention and control schools. Cross-sections will be used because we are measuring the effectiveness of the intervention on the school population and not on individual children. Anthropometric data will be collected at each school by trained members of the research team at baseline (pre GMB1), and after 1, 2 and 3 years (primary endpoint). Height (cm) will be measured using a Charder HM-200P stadiometer and weight (kg) using A&D UC-321 digital scales and BMI (kg/m^2^) will be calculated and compared to the WHO reference population [45].

#### Secondary intervention effectiveness outcomes.

Secondary outcomes include overweight/obesity prevalence of grade 1–6 children, captured at the same four time points, and health behaviours of children in grades 4–6 collected via a self-completed questionnaire on electronic tablets [Samsung Galaxy A9+]. Questionnaires will be completed by classes of children in one school period (~ 45 minutes). The questionnaire will ask about physical activity (self-reported time in moderate to vigorous physical activity, use of active transport to and from school), sedentary behaviour (self-reported screen time for entertainment), diet (intake of vegetables, fruit, water, takeaway foods, sweet snacks, savoury snack and sugar-sweetened beverages), oral health (brushing frequency, oral health, dental care access), sleep (self-reported usual sleep and wake times) and HRQoL (global, physical and psychosocial domains) using validated tools (Table 3) [22,25]. Demographic data will include age, gender, country of birth, and ethnicity. The Index of Community Socio-Educational Advantage (ICSEA) [46] will be used to describe children’s socioeconomic position.

**Table 3:** Primary and secondary outcomes of interests and proposed instrument/measure in supplementary.

Item	Outcome(s) of interest	Proposed Instrument/measure
**Anthropometry** **(objective)**	• Change in z-BMI score (Primary outcome 1) • Change in overweight and obesity prevalence [47]	Height and weight.
**Physical activity and Sedentary behaviour** **(student self-report)**	• Change in the proportion of participants meeting the Australian 24-Hour Movement Guidelines for Children and Young People (5–17) [48] • Change in the percentage of students taking active transport to and/or from school.	Questionnaire containing modified items from the Core Indicators and Measures of Youth Health [49] and SHAPES [50] surveys.
**Diet Type, frequency** **(student self-report)**	• Change in the percentage of participants meeting the Australian Dietary guidelines for fruit and vegetable intake [51] - ◦ Change in intake of serves of fruit ◦ Change in intake of serves of vegetables • Change in frequency of takeaway food consumption • Change in frequency and amount of savoury snacks (i.e., potato chips and savory snacks) • Change in intake of snacks such as lollies and chocolate) • Change in intake of snacks such as cakes, biscuits, sweet pastries and donuts • Change in intake of serves of sugar-sweetened beverages (i.e., soft drinks, sports drinks and energy drinks, cordial, favoured milk) • Change in water intake	Questionnaire containing items generated from the Child Nutrition Questionnaire (CNQ) [52], the Food, Health and Choices Questionnaire (FHC-Q) [53] and the Simple Dietary Questionnaire (SDQ) (Parletta N, Frensham L, Peters J et al (unpublished work)
**Oral Health** **(student self-report)**	• Change in brushing frequency • Change in the rating of oral health • Change in frequency of dentist visits	Questions from the Victorian student health and wellbeing survey [54]
**Health-related quality of Life (HRQoL)** **(student self-report)**	• Change in global health-related quality of life -related quality of life score • Change in subcomponent psychosocial health-related quality of life summary score • Change in subcomponent physical health-related quality of life summary score	Paediatric Quality of Life Inventory (PedsQL) [55]
**Sleep** **(student self-report)**	• Change in self-report sleep time	Bespoke sleep question item based on Berentzen et al [56], where students indicate the times they typically go to bed and wake up on school days.
**Environments (school and community) (school self-report)**	• Change in school policy environment • Change in school physical environment • Change in school economic environment • Change in school socio-cultural environment • Change in committee providing guidance on physical activity and healthy eating • Change in frequency length and attendance at committee meetings • Change in teacher awareness of policies *Nutrition environment at the school:* • Change in food and beverage availability • Change in canteen menu offerings and cost? • Change in nutrition environments • Change in parental support for healthy eating • Change in canteen operations/space for food preparation • Change in student allowance to leave school grounds during the day for food/beverages • Chance in level of priority for nutrition at school • Change in nutrition role modelling • Changes, where applicable in: (1) healthy food choices at reasonable/subsidised price (2) Daily healthy eating specials (3) Healthy eating canteen program (e.g., traffic light labelling system) (4) Routine promotion/ advertisement of healthy food choices (5) Review of the canteen menu on a regular basis (6) Offering healthy food choices during breakfast program (7) Offering healthy food choices in the canteen (8) ‘Nude food’ program or days (9) Stopped the sale of junk foods (10) Held junk food free days (11) Stopped the sale of sugar-sweetened beverages (12) participation in Vic Kids Eat Well (13) participation in Achievement program (14) participation in Stephanie Alexander kitchen garden program (15) Participation in other types of garden program (16) participation in environmental sustainability program related to food (17) Use of food and/or beverage rewards in classrooms (18) Sales of chocolate or lollies as part of fundraising or events for the school (19) Sales of other junk food (e.g., chips, popcorn) as part of fundraising or events for the school. (20) Sales of soda pop or fruit drinks that are not 100% juice as part of fundraising or events for the school (21) Sales of sport drinks as part of fundraising or events for the school (22) Sales of chocolate or lollies as part of fundraising or events for the school (23) Sales of biscuits, cakes, pastries, or other baked goods as part of fundraising or events for the school (24) Sales of fruits or vegetables as part of fundraising or events for the school (25) Sales of 100% fruit juice or vegetable juice as part of fundraising or events for the school (26) Offering cooking classes (27) Offering field trips to fames/primary producers (28) Offering media literacy on special topics related to healthy eating (29) Offering field trips to the local grocery store/farmers markets (30) Offering gardening (e.g., growing produce/school gardens (31) Offering school breakfast program (32) Offering ‘other’ activities (33) Effectiveness of offered activities (34) Barriers to offering activities (35) Allowing students to drink water in the classrooms during class time (36) Number of water fountains on the school grounds (37) allowing students to eat in the classrooms during class time. *Physical activity environment at the school:* • Change in minutes/week/average time devoted to physical activity/organised sport • Change in frequency, length and attendance numbers on relevant committee meetings • Change in how physical activity is characterised • Change in how physical activity is role modelled • Change in how physical activity is promoted at the school • Change in programs/activities related to physical activity • Change in effectiveness of activities	Preschools, Primary Schools: Be Active Eat Well Environment questionnaire [57] and the School Food Environment and Resource Tool (FERST) [58]

#### School-level implementation outcomes.

Each year of the study, the school principal (or nominee) will be invited to complete a school environment audit questionnaire in Qualtrics about their school’s physical activity, healthy eating and well-being policies, practices (i.e., minutes/week devoted to formal physical education/organised sport, canteen menu) (Table 3). In addition, the questionnaire will be used to assess change in frequency, length and attendance numbers of committee meetings providing guidance on the development of policies and practices concerning physical activity, healthy eating, wellbeing and/or mobile phone use (Table 3).

#### Intervention cost-effectiveness.

An economic evaluation will be undertaken to determine whether the STAIR intervention represents ‘value for money’ as compared to usual practice [59]. Intervention costs (financial resources, human resources and equipment) will be estimated using action registers completed by school champions in MS Teams and published sources (including meeting minutes). A within-trial cost-consequence analysis will allow the assessment of intervention costs from the perspective of different stakeholders (schools, partners, limited societal) alongside effectiveness outcomes (primary and secondary outcomes). A modelled cost-utility analysis will extend the costs and effects of the trial over a longer time horizon and across a broader target population and decision context. The ACE-Obesity policy model, a multi-state life table Markov model will then be used to estimate the health benefits (HALYs) and healthcare cost-savings of diseases averted through reduced exposure to BMI because of the intervention [60]. Modelling results will include an assessment of cost-effectiveness using a return-on-investment ratio and costs saved based on healthcare cost-savings of diseases averted over the lifetime of the intervention population. Uncertainty will be incorporated using Monte Carlo simulation using the Ersatz software package [61], and sensitivity analyses will explore the impacts of varying key input parameters on overall cost-effectiveness results. The economic evaluation will follow the recommendations of the Second Panel on Cost-Effectiveness in Health and Medicine [62] and will be reported according to published guidance [63].

#### Implementation outcomes.

As schools will have varying implementation needs, the research team will use a multiple embedded case study design with the intervention school community (n = 14) as the unit of analysis to evaluate the implementation process [64]. Using indicators based on Proctor’s taxonomy of implementation outcomes [65], data will be gathered at baseline (after GMB3 and before GMB4) and after 1, 2, and 3 years (primary endpoint). Participants will be a convenience sample of representatives from the prevention system organisations in the intervention school communities (e.g., school champions, local government, health service, project partners, and researchers). For some outcomes, children will be the unit of analysis. Within group analysis of Implementation outcomes will determine if intervention school communities consider the STAIR implementation support to be effective (acceptable, adopted, appropriate, feasible) [65,66]. Table 4 summarises how and when these implementation outcomes will be measured. Indicators will be finalised with school communities using pragmatic decisions to determine which aspects of implementation will be evaluated and when [67]. Collectively, these outcomes, and a cost-consequence analysis that will disaggregate implementation costs and outcomes, will evaluate the effectiveness of the STAIR implementation process [65–67]. Implementation survey data will be gathered online (Qualtrics) [68] and from meeting minutes, causal loop diagrams and action registers. These will be complemented by key informant interviews analysed thematically in NVIVO.

**Table 4:** Summary of implementation outcomes and indicators.

Implementation outcomes	Indicator/Numerator	Denominator	When measured	Participants	Data Collection Methods
**Acceptability (extent to which organisations are committed to STAIR and satisfied with the implementation process)**	% of schools and partner organisations are satisfied with the STAIR implementation process (Primary outcome 2)	All intervention schools and partner organisations in local government area	Baseline, year 1,2,3	Intervention school & partner organisation representatives	Implementation survey Key Informant Interview (KII) Action Register
**Adoption (extent to which organisations adopt STAIR intervention actions)**	% of school and partner organisations delivering STAIR intervention actions (Primary outcome 3)	All intervention schools and partner organisations in local government area	Baseline, year 1,2,3	Intervention school & partner organisation representatives	Implementation survey KII Action Register
	% of school and partner organisations with formal partnerships to deliver STAIR intervention actions (Primary outcome 3)	All intervention schools and partner organisations in local government area	Baseline, year 1,2,3	Intervention school & partner organisation representatives	MOUs
**Appropriateness and feasibility (perceived and actual fit of STAIR with organisations)**	% of school and partner organisations ready to implement STAIR implementation strategies (also a measure of adoption at baseline)	All intervention schools and partner organisations in local government area	Baseline	Intervention school & partner organisation representatives	Organisational readiness survey
	% of school and partner organisations who agree implementation functions are useful/relevant	All intervention schools and partner organisations in local government area	Year 1,2,3	Intervention school & partner organisation representatives	Implementation survey
	% of staff trained in STAIR implementation strategies (prioritised actions from GMB1–3)	All relevant staff in schools and partner organisations	Year 1,2,3	Intervention school & partner organisation representatives	Implementation survey
	% of prevention system organisations offering incentives for staff to work on STAIR implementation strategies	All intervention schools and partner organisations in local government area	Years 1,2,3	Intervention school & partner organisation representatives	Implementation survey Action register
	% of staff participating in GMB 5 + workshops	All relevant staff in intervention schools and partner organisations in local government area	Years 1,2,3	Intervention school & partner organisation representatives	Workshop records
**Fidelity (extent to which intervention actions delivered as intended)**	% of prevention and implementation strategies with high leverage (measured using Public Health 12) [69]	All prevention and implementation strategies	Baseline, year 1,2,3	Researcher team	Action Plan and GMB maps, school environmental audit
	% of baseline intervention strategies remaining at 1, 2 and 3 years	Number of intervention strategies in original action plan	Year 1,2,3	Researcher team	Action Plan and GMB maps
	% new intervention strategies at 1, 2 and 3 years	Number of intervention strategies in original action plan	Year 1,2,3	Researcher team	Action Plan and GMB maps
**Penetration (extent to which prevention system organisations are involved)**	% of schools with a school champion	All intervention schools	Year 1,2,3	Intervention school representatives	Implementation survey KII
	% of schools providing healthy eating or physical activity opportunities	All intervention schools	Year 1, 2, 3	Intervention school representatives	Implementation survey
	% of schools participating in the study	Total number of schools selected to participate study	Baseline year 1, 2, 3	Research team	Project records
	% of prevention system organisations who are members of the leaderships team	All prevention system organisations in local government area	Baseline year 1, 2, 3	Intervention school & partner organisation representatives	Leadership team membership and meeting minutes
	% staff time (including volunteers) dedicated to implementation of STAIR intervention actions	Total staff time in organisation	Year 1, 2, 3	Intervention school & partner organisation representatives	Implementation survey Duty statements
	Prevention system organisations budget allocated to STAIR intervention actions	Total budget of organisation	Year 1, 2, 3	Intervention school & partner organisation representatives	Implementation survey Action Register
	Number of times STAIR appears in local media	Frequency of local media publications	Year 1, 2, 3	Research team	Local media publications
	% of schools who have formed an implementation team	All intervention schools	Year 1, 2, 3	Intervention school representatives	Research team
**Sustainability (extent to which organisations remained engaged)**	% of prevention system organisations involved at 3 years	Number of organisations involved at baseline	Year 3	Intervention school & partner organisation representatives	Meeting minutes KII
	Integration of STAIR intervention strategies into prevention system organisation mission at 3 years	Organisational mission at baseline	Year 3	Intervention school & partner organisation representatives	Implementation survey Organisation website
**Implementation cost (cost of implementation)**	Cost for schools and partners	All schools and partners	Baseline, Year 1, 2, 3	Intervention school & partner organisation representatives	Administrative records, tracking active implementation functions
	% of prevention system organisations who feel confident that intervention strategies represent good value for money	All schools and partners	Baseline, Year 1, 2, 3	Intervention school & partner organisation representatives	Implementation survey
	% of prevention system organisations who feel confident that they have the resources needed to implement STAIR	All schools and partners	Baseline, Year 1, 2, 3	Intervention school & partner organisation representatives	Implementation survey

### Sample size calculations

Victorian Government Department of Education summary statistics compiled in 2023 indicate that, in the 21 regional and remote LGAs of the Southwestern Department of Education region (excluding schools on the geographical fringe of Melbourne in Brimbank, Melton, and Western Melbourne education areas), there were 57,828 children in primary schools. A total of 47,087 (81.4%) children attended the 147 eligible schools with an average of 320 students per school. By recruiting 28 schools and accounting for a 20% school dropout rate, it is anticipated that data will be collected across all time points (baseline, year 1, year 2 and year 3) in 22 schools. A sample of 22 schools, with approximately 200 participating students per school, will provide 83% power to detect a difference of 0.13 units in the primary outcome (z-BMI) between study groups; assuming z-BMI standard deviation 1.1 and intra-cluster correlation coefficient 0.02, as found in the WHO STOPS trial in a comparable population [22,70]. An effect size of 0.13 units in z-BMI it was shown to be achievable, using similar intervention methods, after 2 years in the WHO STOPS trial. Power was estimated using Shiny CRT calculator based on a cluster randomised parallel design with baseline measure, cross-sectional samples, and exchangeable correlation structure (α = 0.05) [71].

### Statistical analysis

Students will be the unit of observation for effectiveness analyses and analyses will be conducted under the intention-to-treat principle. The effect of the STAIR intervention on the z-BMI will be assessed using a linear mixed model with study group, time (baseline, year 1, year 2, year 3), and the interaction group × time as fixed effects. School and student will be included as random effects to account for clustering of students within schools and repeated measures in students measured more than once). Secondary outcomes will be analysed by fitting a generalised linear mixed model with link and distribution selected according to the outcome distribution. Whether age modifies the intervention effect will be explored using the same modelling approach including additional interaction terms. The environmental audit, and implementation outcomes will be analysed at the school level using descriptive statistics and thematic analyses.

### Data management

Data custodianship arrangements and rights of access, rights to analyse, use and re-use the data by Deakin University and the collaborating institutions is covered by the Multi-Institutional Agreement between participating institutions. Information obtained in connection with this research that can identify participants will remain confidential. All information provided will be used for research purposes only, it will be collected and kept in confidence and will not be released to anyone outside the study team. Hardcopies of data will be kept in locked facilities at Deakin University in accordance with government guidelines. Digital copies of data will be kept in a secure Deakin University server. Data will be added to a mediated access repository on a secure DEAKIN server until such time as it is requested by other potential users, at which point de-identified data will be transferred to the other researchers via Deakin University approved software.

All data collected from children will be disposed of after a minimum period of 15 years after the child reaches 18 years of age and all other data after a minimum period of 7 years from the last publication. Only members of the study team will have access to the data/records and the computer records will be password protected, to strict security standards. In any publication, presentation, or discussion the information will be provided in such a way that any child cannot be identified. The research team will not report individual child measurements. Results are reported at an aggregate level so no individual child can be identified. In reports back to schools and partners, the results are presented at an aggregate (e.g., Year 4 girls, All Year 6 children). The research team will not share individual-level data with any individual or agency other than the listed researchers on this paper (e.g., No individual-level data will be shared with a Partner Organisation, only summary reports at an aggregate level).

### Safety considerations

It is recognised that participating in research may impact children’s safety, psychological security and wellbeing. The research team will take the following precautions to protect children’s safety, emotional and psychological health and wellbeing:

1. Height and weight measurements will be taken in private measuring booths, so students cannot see one another, and their privacy is protected.
2. All measurements will be taken in silence, the weight scale will be physically blocked from students’ view. Students are only told their height/weight measurements if they ask.
3. All measurements are voluntary, and students can participate as much or as little as they like. Students are told on data collection days that overall participation and each component is voluntary even if an opt-out form has not been received. They can participate as much as they like (e.g., just do the questionnaire, just have their height measured). Prior to anthropometric measurements assent is sought with the student “Is it okay if I measure your height?”, “Is it okay that I measure your weight?”
4. Researchers will look out for students that look uneasy, anxious or embarrassed and make rational decisions whether to measure the height and weight of these students. When such instances occur, data collectors either complete the measurement they are taking and skip the next one (e.g., finish measuring height and skip measuring weight) or direct the child to the next activity (e.g., questionnaire) or back to class.
5. Each school is required to provide a registered teacher (typically the classroom teacher) to oversee measurements whilst researchers are present. School teachers will also look out for distressed students and notify the researchers if they think that participation would not be in the best interest of the child. If a schoolteacher thinks a child should not participate, the research team will not measure weight and height or complete the questionnaire.
6. The research team will provide the number for support resources in the PLS
7. All data collectors will have a current Victorian Working with Children’s Check or equivalent (e.g., Registered Teacher).
8. All staff will be aware of obligations under the Victorian Child Safe Standards regarding mandatory reporting.

### Timeline

Fig 2 shows the proposed timeline for the project. Due to delays in obtaining approval from the Department of Education, the project has been delayed and as such no data has been collected. It is anticipated data collection will begin in April 2025 and final data will be available in 2029.

### Reporting and dissemination

This protocol and results will be reported following the CONSORT statement for reporting cluster randomised controlled trials, the Consolidated Health Economic Evaluation Reporting Standards (CHEERS) checklist for reporting the economic evaluation and, the Standards for Reporting Implementation Studies (StaRI) statement.

After each round of data collection, schools and partners will be provided with co-designed presentations and reports to keep them informed of progress and outcomes. The results of STAIR will also be disseminated via academic publications, conference presentations, and publications linked to postgraduate research projects.

## Discussion

Ending childhood obesity is a global and national priority [2]. STAIR will be the first real-world study to combine systems thinking concepts for co-designing intervention strategies with active implementation strategies to help schools sustain them. It aligns with children’s right to health and is based on the best available evidence for effective obesity prevention [15]. Furthermore, by measuring the impact of intervention strategies on a range of health outcomes of relevance to Australian children (such as HRQoL, physical activity and oral health), we are recognizing that interventions can and should perform multiple duties [72]. As such, outcomes will add to existing knowledge on *what* intervention actions work, by providing evidence on *how* to implement overweight/obesity prevention strategies, *who* is responsible for the implementation strategies, *where* accountability for implementation sits and *how much* the anticipated health gains will cost [73]. Collectively these outcomes will provide school communities, partners and government with direct insight into how to prevent childhood overweight/obesity in school communities, how much it will cost and how best to support communities with ongoing efforts. As findings become available during the trial, STAIR’s research partners will ensure they are translated into local and state-level policy and practice.

### Strengths and limitations of this study

- We will leverage current knowledge, use a co-design intervention process and will apply a cost-effective type-2 hybrid design to provide evidence on whether, and how intervention impact can be sustained in the longer term.
- STAIR will be the first real-world study to combine systems thinking with active implementation to help schools create and sustain healthy environments. The study will provide evidence on what intervention actions work, how to implement these actions, who is responsible for the implementation strategies, where accountability for implementation sits and how much the anticipated health gains will cost.
- Participating schools may be comparable to schools in other rural and regional areas but will not be representative of all Victorian schools.
- Schools and researchers will not be blinded to intervention allocation

## Supporting information

S1 Fig SPIRIT checklist. (PDF)
